# Self-Diagnosis of SARS-CoV-2 from Saliva Samples at Home: Isothermal Amplification Enabled by Do-It-Yourself Portable Incubators and Laminated Poly-ethyl Sulfonate Membranes

**DOI:** 10.3390/diagnostics14020221

**Published:** 2024-01-19

**Authors:** Sergio Bravo-González, Everardo González-González, Valeria Perales-Salinas, Iram Pablo Rodríguez-Sánchez, Jose E. Ortiz-Castillo, Adriana Vargas-Martínez, Victor H. Perez-Gonzalez, Claudia Maribel Luna-Aguirre, Grissel Trujillo-de Santiago, Mario Moisés Alvarez

**Affiliations:** 1 Centro de Biotecnología-FEMSA, Tecnologico de Monterrey, Monterrey 64849, NL, Mexico; a01191795@tec.mx (S.B.-G.); e.gzz@tec.mx (E.G.-G.); v.peralessalinas@westernsydney.edu.au (V.P.-S.); maribel.luna@tec.mx (C.M.L.-A.); 2 Departamento de Bioingeniería, Tecnologico de Monterrey, Monterrey 64849, NL, Mexico; 3 Laboratorio de Fisiología Molecular y Estructural, Facultad de Ciencias Biológicas, Universidad Autónoma de Nuevo León, San Nicolás de los Garza 66455, NL, Mexico; iramrodriguez@gmail.com; 4 Alfa Medical Center, Guadalupe 67100, NL, Mexico; 5 Departamento de Ingeniería Mecátrónica y Eléctrica, Tecnologico de Monterrey, Monterrey 64849, NL, Mexico; eric_ortiz95@hotmail.com (J.E.O.-C.); adriana.vargas.mtz@tec.mx (A.V.-M.); vhpg@tec.mx (V.H.P.-G.)

**Keywords:** saliva, SARS-CoV-2, COVID-19, diagnostic, portable, isothermal nucleic acid amplification, extraction free, at home, LAMP

## Abstract

COVID-19 made explicit the need for rethinking the way in which we conduct testing for epidemic emergencies. During the COVID-19 pandemic, the dependence on centralized lab facilities and resource-intensive methodologies (e.g., RT-qPCR methods) greatly limited the deployment of widespread testing efforts in many developed and underdeveloped countries. Here, we illustrate the development of a simple and portable diagnostic kit that enables self-diagnosis of COVID-19 at home from saliva samples. We describe the development of a do-it-yourself (DIY) incubator for Eppendorf tubes that can be used to conduct SARS-CoV-2 detection with competitive sensitivity and selectivity from saliva at home. In a proof-of-concept experiment, we assembled Eppendorf-tube incubators at our home shop, prepared a single-tube mix of reagents and LAMP primers in our lab, and deployed these COVID-19 detection kits using urban delivery systems (i.e., Rappifavor or Uber) to more than 15 different locations in Monterrey, México. This straightforward strategy enabled rapid and cost-effective at-home molecular diagnostics of SARS-CoV-2 from real saliva samples with a high sensitivity (100%) and high selectivity (87%).

## 1. Introduction

The limitations of the currently available strategies for conducting widespread diagnostics became clear during the COVID-19 pandemic [1,2,3]. A wide spectrum of SARS-CoV-2 detection methods was implemented to increase diagnostic coverage. For example, lateral flow immunoassays (LFIAs) have become relevant for identifying the presence of SARS-CoV-2 or antibodies produced against SARS-CoV-2 in a rapid and cost-effective manner [4]. LFIA methods to detect SARS-CoV-2 antibodies remain useful for determining whether a person was exposed to SARS-CoV-2 weeks or months before testing, but they are not useful for identifying infective individuals. LFIA methods based on the detection of antigens (i.e., SARS-CoV-2 proteins) identify infective subjects but have a lower accuracy and reproducibility than molecular methods [5,6]. Therefore, the reference methods for the diagnosis of infection by SARS-CoV-2 are based on RT-qPCR protocols [2,3,7,8], which possess unchallenged detection thresholds, sensitivity, and selectivity.

**Figure 1:**
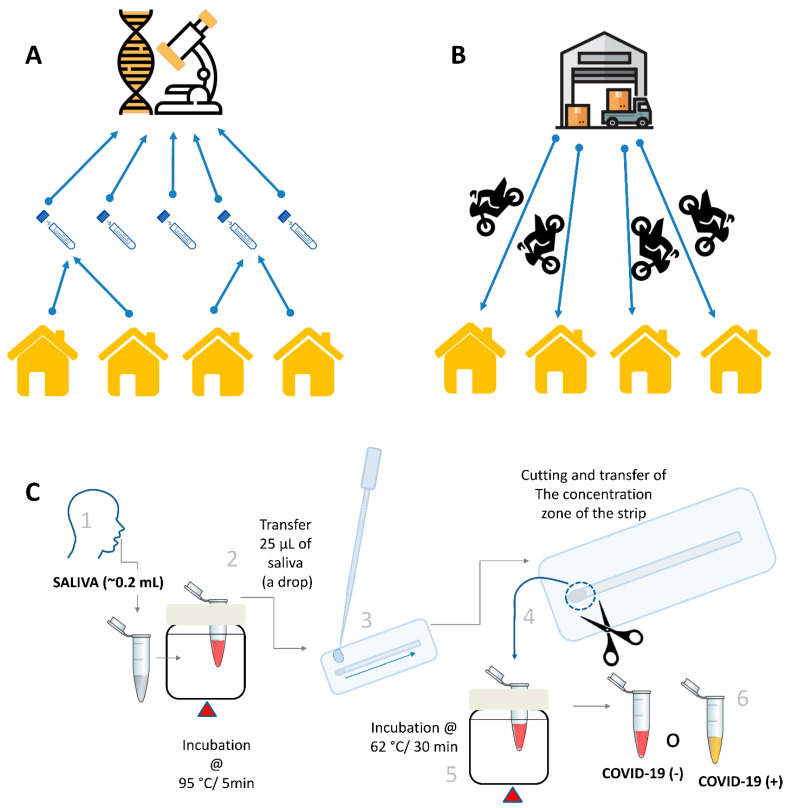
From conventional testing to decentralized and parallelized point-of-care diagnostics of infectious diseases. (**A**) Schematic representation of a conventional strategy for testing for infectious diseases: samples (i.e., mainly nasopharyngeal) are taken by health care personnel and sent to a centralized lab for molecular analysis (i.e., mainly by PCR-based methods). (**B**) Alternatively, portable diagnostic kits can be delivered (using Rappifavor or Uber services) from a central warehouse to the point of diagnosis for self-collection and self-diagnosis. (**C**) Schematic representation of the algorithm of self-diagnosis from saliva samples using LAMP reagents, a portable incubator, and a laminated PES membrane for nucleic acid concentration. The algorithm includes (1) self-collection of saliva, (2) thermal deactivation of viral particles, (3) nucleic acid concentration in PES membrane devices, (4) cutting and transfer of the circular area of the PES device to the LAMP reaction mix, and (5) LAMP reaction during isothermal incubation, and (6) visual determination of results by color change from red to yellow. Icons marked with asterisks were designed by Freepik: warehouse icon from Toempong linear color, and microscope icon from Eucalypt.

However, to achieve a high sensitivity and selectivity, q-PCR depends on centralized lab facilities, costly and poorly portable equipment, expert technicians, and RNA extraction methodologies that also need central labs and dedicated expertise (Figure 1A) [2,3,7]. These requirements severely limited widespread testing for COVID-19 in developed and developing economies around the globe [1,3,9].

COVID-19 has served as a cruel invitation to develop and implement true point-of-care systems that can be easily deployed during emergencies for widespread testing [10]. Our hope is that our worldwide collective experience will allow for a drastic transformation of the ways in which we conduct diagnostics for infectious diseases.

One idea is to reverse the prevailing system of the diagnosis of respiratory infections (based on receiving nasopharyngeal samples in a central lab for testing by expert technicians) (Figure 1A) and instead to engineer [7] a decentralized strategy (consisting in sending compact and portable diagnostic kits from a warehouse for personal testing at home by the subject from self-obtained saliva samples) as shown in Figure 1B.

Take the detection of SARS-CoV-2, the causal agent of COVID-19, as a model. Conducting SARS-CoV-2 testing from saliva would greatly simplify sampling and would enable the intensification of the COVID-19 testing efforts [11,12,13,14,15]. Moreover, viral loads in saliva may be higher or comparable than in nasopharyngeal samples [16,17,18].

The present contribution introduces a SARS-CoV-2 diagnostic system based on loop-mediated amplification (LAMP) that is enabled by a do-it-yourself (DIY) portable incubator and a laminated PES membrane. Recent reports have demonstrated that LAMP-based methods [19,20,21] may be comparable to PCR in terms of accuracy (i.e., limits of detection between 10 and 100 copies per reaction, sensitivity above 95%, and selectivity above 90%) but superior in terms of outreach [3,21]. This proof-of-concept pilot study illustrates the implementation of a cost-efficient strategy for the rapid fabrication of testing kits that can be easily deployed to enable the diagnosis of infectious diseases at home.

## 2. Materials and Methods

We describe the contents of a portable diagnostic kit that enables saliva SARS-CoV-2 testing at home, and we review each of the conceptual stages of this diagnostic scheme, including saliva sampling, thermal inactivation, sample concentration, and amplification.

### 2.1. Development of LAMP-Based Portable Kits for SARS-CoV-2 Identification

**Figure 2:**
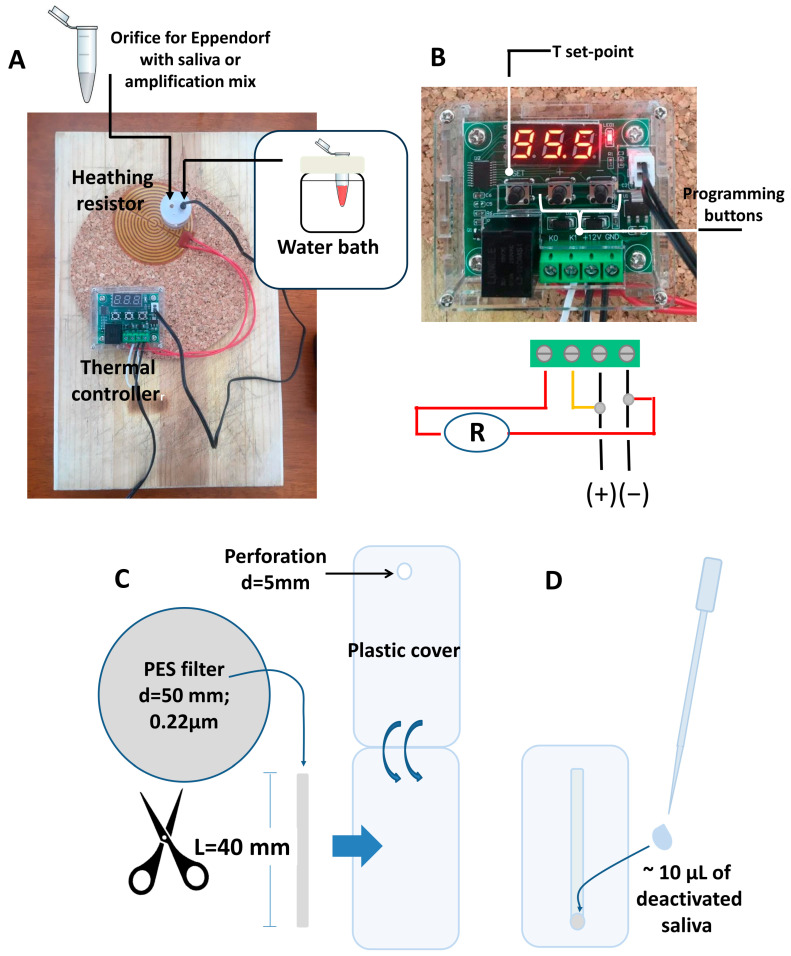
Portable DIY kit for SARS-CoV-2 identification in saliva samples. (**A**) Actual image of the incubation system used for thermal inactivation of SARS-CoV-2 from saliva samples and incubation of the LAMP reactions. (**B**) Close-up view of the commercial thermostat used for the inactivation and incubation of saliva samples and schematic representation of the wiring of the system to the power source and the heating mat. (**C**) Schematic representation of the laminated PES membrane used for nucleic acid concentration (top view). (**D**) A drop of saliva is deposited on the exposed circular region of the PES device.

We assembled nearly 30 diagnostic kits for SARS-CoV-2 diagnostics from saliva during the extended quarantine related to COVID-19 pandemics in Monterrey, México, from May 2020 to January 2021. These kits were sent to volunteers located in different locations in the city of Monterrey, Mexico (a metropolitan area with nearly 5,500,000 inhabitants), to enable home diagnostics of SARS-CoV-2 from self-collected saliva samples. Each kit consisted of (a) a portable incubator for Eppendorf tubes and reagents, (b) materials required for the sampling, handling, and concentration at home, and (c) reagents for a LAMP-based amplification (i.e., reaction mix and primers) (Figure 2).

We used commercial reagents (LAMP Warm-Mix from New England Biolabs; Ipswich, MA, USA) and proprietary primers whose sequences are provided in Table 1. We estimate the overall cost of this kit, including reagents and supplies, at USD 30. All materials are available at https://www.amazon.com (accessed on 12 December 2023) and other established e-commerce distributors.

### 2.2. Fabrication of Portable Isothermal Incubators

We assembled 10 simple Eppendorf-tube incubators (Figure 2A) suitable for saliva inactivation and amplification in a small shop established at the home of one of the authors. These incubators integrated a W1209 12 V DC thermo-controller (from MakerHawk, https://www.amazon.com (URL accessed on 12 December 2023); Shenzhen, China), a 12 V round and flexible heating plate (from Icstation, 12 V, 13 W, 70 mm, round polyamide; https://www.amazon.com (accessed on 12 December 2023), Shenzhen, China), a 12 V and 1 A power adapter from AC 100–240 V to DC 12 V (e.g., N/X 12 V adapter; https://www.amazon.com (accessed on 12 December 2023), Shenzhen, China), and a 20 mL glass jar with a silicone lid (from Juvale; https://www.amazon.com (URL accessed on 14 January 2021), Monrovia, CA, USA). Three orifices were produced in the silicone lid using a perforating tool (e.g., a hole-punching machine; WoneNice belt hole puncher; available at https://www.amazon.com (accessed on 12 December 2023), Shenzhen, China). Two of these orifices should be of sufficient diameter (~5 mm) to allow for the complete insertion of 200 µL Eppendorf tubes. The second orifice allows for the depressurization of the water bath when filled and fully closed. When in use for heating, the glass container should be filled with water, the silicone lid should be hermetically closed, and the thermocouple of the thermal controller should be fully inserted into the water bath.

Figure 2A shows the assembled incubator; Figure 2B shows a scheme indicating how the heating element (heating resistance) and the 12 V power adapters should be connected. These portable incubators were part of the diagnostic kits we sent to different locations in Monterrey, NL, using urban delivery services (Rappifavor, Mexico City, Mexico; or Uber, Mexico City, Mexico).

### 2.3. Materials for Saliva Sampling and Concentration

The portable diagnostic kit included a set of materials to facilitate the operations related to the collection, handling, and concentration of real saliva samples at home. This set consisted of a container for self-collection of saliva samples (i.e., a small glass container with a hermetic silicone lid or a sterile plastic container), an empty 200 µL Eppendorf tube to be used for the inactivation of saliva, two disposable 100 µL pipettes for saliva transfer, and a lab-made concentrating device (Figure 2C) made of a laminated PES filter membrane.

**Figure 3:**
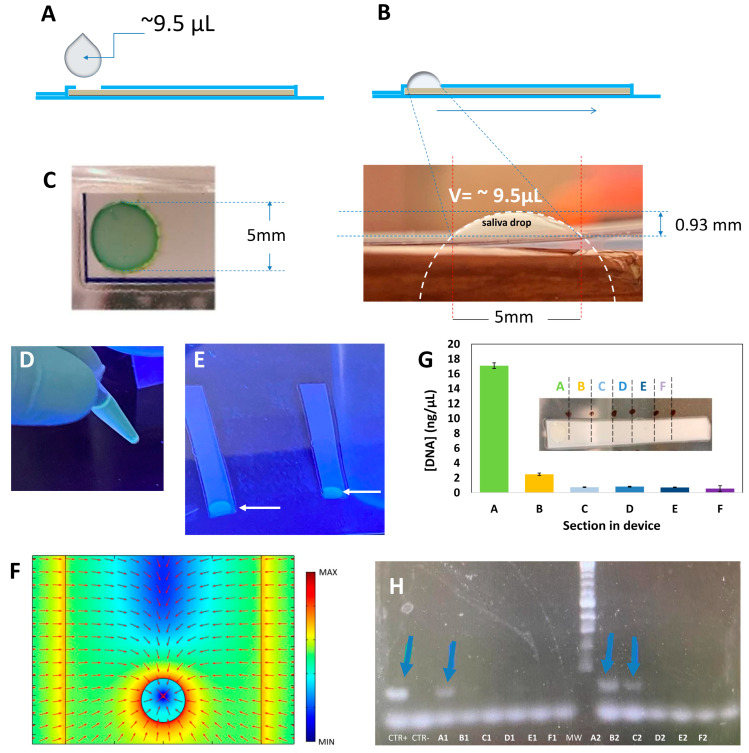
Characterization of the process of concentration of nucleic acids from saliva in laminated PES devices. (**A**) Schematic representation of a laminated PES strip (lateral view). A stripe of PES filter (yellow) was laminated using commercial plastic films (blue). (**B**) Estimation of the volume of a drop of saliva, as deposited in the PES device. The volume of the drop was calculated using geometric arguments. (**C**) The liquid component of the drop of saliva is absorbed and driven by capillarity through the PES membrane strip, while nearly 90% of the charged molecules in the solid fraction are retained in the exposed circular region as illustrated by the deposition of a drop of dyed green solution. (**D**) SARS-CoV-2 genetic material suspended in PBS and stained with a fluorescent DNA intercalating reagent (EvaGreen Dye) for visualization. (**E**) Drops of suspensions of stained SARS-CoV-2 genetic material deposited in the exposed circular region of the laminated PES devices. Exposure to UV light suggests that the genetic material mostly remains confined within the exposed region of the PES device where originally dispensed (indicated with a white arrow). (**F**) Simulations of the electrostatic force field acting on negatively charged genetic material in the vicinity of the exposed circle in PES devices. A circle of high electric charges (red rim) develops in the perimeter of the exposed PES surface. This highly charged rim may act as an electrostatic barrier that retains the negatively charged material within the exposed circular area of the PES surface. (**G**) Concentration of SARS-CoV-2 synthetic DNA trapped in different sections of the PES devices as determined using spectrophotometric techniques. The inset shows the different segments into which laminated PES strips were divided for DNA determinations. (**H**) SARS-CoV-2 synthetic DNA eluted from the different sections of laminated PES strips as observed by gel electrophoresis. Blue arrows highlight the amplification of SARS-CoV-2 synthetic DNA in different sections of PES device.

The fabrication of the laminated PES membrane devices required for the concentration of genetic material in the saliva samples is graphically described in Figure 2C. Rectangular strips of PES filter paper (5 × 0.5 cm) were cut from circles of PES membrane filters (Allpure, available at https://www.amazon.com (accessed on 12 December 2023), Auburn, WA, USA). Specifically, we used a PES membrane with a 0.22 µm pore size and a 50 mm diameter. The PES membrane strips were laminated with lamination film (from GBC, select-a-size thermal lamination roll, for 9″ and 12″ laminators; available at https://www.amazon.com (accessed on 12 December 2023), Lake County, IL, USA) using a portable laminating machine (GBC P4377, slim thermal laminator machine, 9″; available from https://www.amazon.com (accessed on 12 December 2023), Lake County, IL, USA). The strips were laminated, leaving an exposed 5 mm diameter circle on one face (Figure 2C and Figure 3A). This was performed using a paper-cutting machine (Silhouette Portrait paper cutter, from Silhouette; available at https://www.amazon.com (accessed on 12 December 2023), Lindon, UT, USA) to cut 5 mm diameter circles in only one side of the lamination film.

A computational 3D stationary study was conducted in COMSOL Multiphysics 5.2 (COMSOL, Inc., Burlington, MA, USA) to qualitatively model the electrostatic force field within the laminated PES membrane, leading to the concentration of genetic material. The model considered a positive surface charge distribution over the PES membrane (with an excess of positive charges at the exposed region). The laminating film was given a negative charge at the interface with the PES membrane and a positive surface charge distribution on the outer faces. The excess positive charges at the PES exposed region and the positive charge distribution at the laminating film’s outer surface are due to the triboelectric effect that takes place during lamination [22]. A negative charge was assumed for the genetic material [23].

### 2.4. Amplification Reagents

We used commercial amplification reagents (LAMP Warm-Mix from New England Biolabs; Ipswich, MA, USA) and proprietary primers (sequences are provided in Table 1). The commercial reaction mix contains phenol red, a widely used pH indicator, which changes in color from red to yellow at pH 6.8 [20]. During LAMP, the pH of the reaction mix continuously changes from neutral to acidic values as protons are produced [24,25]. This release of hydrogen ions is quantitative [20]. The production of H+ is high, since it is quantitatively proportional to the number of newly integrated dNTPs [26]. A proprietary primer set (set β) was used (Table 1) to define the amplification region within sequences of RNA encoding for protein N of SARS-CoV-2. This consensus sequence is based on the analysis of alignments of the SARS-CoV-2 sequences of the N gene reported from December 2019 to March 2020.

To facilitate home experiments and to enable a one-pot reaction scheme, we prepared single-tube mixes of LAMP-based reagents and primers in our lab at Tecnologico de Monterrey. We used standard 200 µL PCR-Eppendorf tubes containing 24 µL of the reagent and primer mix. Each tube contained 12.5 μL of commercial readymix (WarmStart^®^ Colorimetric LAMP 2× Master Mix (DNA & RNA) from New England Biolabs; Ipswich, MA, USA), 4.4 µL of proprietary primers (1.6 μM FIP primer, 1.6 μM BIP primer, 0.2 μM F3 primer, 0.2 μM B3 primer, 0.4 μM LF primer, 0.4 μM LB primer), and nuclease-free water to the final reaction volume of 24 μL. The commercial readymix contains phenol red as a pH indicator to reveal the change in pH during LAMP across the threshold of pH = 6.8.

### 2.5. Saliva Sampling, Inactivation, and Concentration

Real saliva samples were self-collected by 10 volunteers who were experiencing symptoms presumably related to respiratory diseases and had agreed to participate in this study by signing an informed written consent that described the study. The collection was considered and described under an experimental protocol approved on 20 May 2020 by the institutional committee of Alfa Medical (Research Committee; resolution AMCCI-TECCOVID-001).

Saliva samples (200–500 µL) were collected in small glass or plastic containers included in the diagnostic kit for this purpose (as described). A volume of approximately 150 µL of each saliva sample was then transferred to an empty Eppendorf tube using a disposable pipette. Eppendorf tubes containing saliva were inserted into one of the orifices of the incubator silicone lid so that the saliva sample was completely immersed in the water bath. The saliva samples were incubated at 95 ± 4 °C for at least 5 min for inactivation (i.e., to inactivate viral particles, denature proteins, and enzymes and release RNA content from the viral capsid). A recent report recommends an extended heating period at 95 °C for 30 min [13], which can also be achieved with our DIY portable incubator. To do this, the temperature setting of the thermo-controller was set at 97.5 °C, and the tolerance setting of the controller P1 was set at 0.1.. This program reactivates the heating when the temperature drops below 96.5 °C.

After incubation, one drop of saliva was taken from the bulk of the liquid and transferred to the laminated PES membrane device using a disposable pipette. The drop of saliva should be deposited on top of the exposed circular section of the PES membrane strip and left to rest for approximately 20 min to allow for the absorption of the excess liquid by the strip.

### 2.6. Amplification Protocol

After absorption, the region of the laminated strip containing the disk should be cut using a disposable razor blade and deposited into the 24 µL reaction mix contained in the 200 µL Eppendorf tube. The LAMP experiments were conducted using the DIY portable Eppendorf-tube incubators (Figure 2A) operated at a constant temperature in the range of 60 to 65 °C for 30 min.

We used a LAMP protocol consisting of a single-stage incubation at 62.5 ± 2 °C for 30 min. To do this, the temperature setting of the thermal controller was set at 62.5 °C (Figure 2A), and the tolerance setting of the controller (P1) was set at 0.1. This program reactivates the heating when the temperature decreases below 62.4 °C. Since the commercial reaction mixture contained phenol red, a change in color from red to yellow indicated that the amplification process had proceeded to an extent that allowed for the pH of the reaction mix to cross the pH threshold of the indicator (pH ~6.8) and the sample should be presumed positive.

### 2.7. Documentation of Amplification Products

The amplification products from the PCR and LAMP assays were documented using conventional gel electrophoresis techniques. In these experiments, we analyzed 10 μL of the LAMP or PCR product by 1.2% agarose electrophoresis in tris-borate-EDTA buffer (TBE). The Quick-Load Purple 2-Log DNA Ladder (New England Biolabs, Ipswich, MA, USA) was used as a molecular weight marker. Gels were dyed with Gel-Green from Biotium (Fremont, CA, USA) using a 1:10,000 dilution.

### 2.8. Preparation of Non-Infective Positive Samples

We used commercial plasmids containing the complete N gene from SARS-CoV-2 (Integrated DNA Technologies, Coralville, IA, USA) as positive controls and to spike real saliva samples. Samples that contained different concentrations of synthetic SARS-CoV-2 nucleic acids (from 1 × 10^4^ copies µL^−1^ to 10 copies mL^−1^ of saliva) were prepared by successive dilutions from an original stock that contained 200,000 copies µL^−1^.

An additional set of non-infective positive samples was prepared by spiking real saliva samples with RNA extracts from human volunteers. We used 5 samples of RNA extracts from COVID-19 positive persons, as determined by RT-PCR analysis. The de-identified samples were kindly donated by Hospital Alfa, Medical Center, Guadalupe, Nuevo León, México. Nasopharyngeal samples had been collected from two patients after obtaining informed and signed written consent and in complete observance of good clinical practices, the principles stated in the Helsinki Declaration, and applicable lab operating procedures at Hospital Alfa. Every precaution was taken to protect the privacy of the sample donors and the confidentiality of their personal information. The experimental protocol was approved on 20 May 2020 by a named institutional committee (Alfa Medical Center, Research Committee; resolution AMCCI-TECCOVID-001). RNA extraction and purification were conducted at the molecular biology laboratory at Hospital Alfa. The Qiagen QIAamp DSP Viral RNA Mini kit was used for RNA extraction and purification by closely following the directions of the manufacturer.

### 2.9. PCR Assays

A group of 11 anonymous volunteers who had experienced symptoms presumably related to respiratory diseases granted consent to participate in this study and disclose the results of their RT-qPCR diagnostics. These volunteers received a diagnostic kit at home and conducted a presumptive self-diagnostic test using the LAMP-based diagnostic kit. The kits were distributed to volunteers using available urban delivery services in Monterrey (i.e., Rappifavor or Uber). These volunteers underwent clinical RT-qPCR diagnosis as well. The RT-qPCR assays were performed in three different laboratories in Monterrey, México (i.e., Hospital San José, Tecnológico de Monterrey; Alfa Medical; and Laboratorio Salud Digna). To our knowledge, each of these laboratories used variations of the protocols recommended by the US Centers for Disease Control for the standard diagnostics of COVID-19 (i.e., N1 assay) using RT-qPCR. These protocols were approved by the Secretaría de Salud de México for use in the diagnosis of SARS-CoV-2.

## 3. Results

### 3.1. Overall Description of the Diagnostic Algorithm

Here, we show the feasibility of using a simple molecular method for the diagnosis of COVID-19 at home from self-collected saliva samples. We integrate the steps of the self-collection of saliva samples, the use of thermal inactivation at 95 °C for 5 min (an inactivation protocol reported previously) [14,27], the concentration of the saliva samples using simple paper filter devices, and the use of colorimetric LAMP for the diagnosis of SARS-CoV-2 (Figure 1C). After self-collection at home, a small volume of saliva was heated at ~95 °C for 5 min to inactivate SARS-CoV-2 (and other pathogens and enzymes potentially present in the saliva sample). A drop of thermally deactivated saliva was then placed in a small micro-well fabricated in a laminated microfluidic poly-ethyl sulfonate (PES) membrane device. As discussed later, this simple concentration strategy allows for the recovery of most of the genetic material contained in 10 to 25 µL of saliva. Cutting out the section of the filter paper device where the nucleic acids are concentrated and adding this piece to a colorimetric LAMP reaction mix enables the amplification of SARS-CoV-2 RNA. If the amplification proceeds, an associated change in the color from red to yellow will be observed in positive samples.

### 3.2. Assessment of Sensitivity and Specificity Using Artificial Samples

Before using this diagnostic strategy for real samples, we conducted an extensive number of diagnostic assays at home (*n* = 100) to establish the limits of detection, the sensitivity, and the specificity of the LAMP-based diagnostic strategy described here. To perform this, we prepared non-infective samples containing different amounts of synthetic SARS-CoV-2 genetic material in our lab (as described in the Materials and Methods). Fifty of these samples contained 10,000 copies of synthetic SARS-CoV-2 genetic material in 25 µL of the reaction mix (i.e., equivalent to 10 × 10^6^ copies mL^−1^). In this set, we were able to correctly identify all samples containing SARS-CoV-2 material (i.e., a sensitivity of 100%). In all positive samples, the change in color was clearly observed within a time frame ranging from 10 to 25 min.

The sensitivity of this LAMP reaction decreased in samples containing fewer synthetic copies of SARS-CoV-2 genetic material. In an analogous experiment, we analyzed 46 samples containing 1000 copies of synthetic SARS-CoV-2 genetic material. In this case, the sensitivity of the method, as implemented at home, decreased to 93.1%. Similarly, in 50 samples that contained 100 copies of synthetic SARS-CoV-2 genetic material, we observed a selectivity of 90.0%. However, in samples containing 10 copies of synthetic SARS-CoV-2 genetic material, we observed a sensitivity of 40%. Since some samples that did not contain SARS-CoV-2 genetic material were identified as positive (3/50), the specificity of the assay was 94%. These results suggest that this portable method of detection can reliably amplify 100 or more copies of artificial SARS-CoV-2 genetic material with accuracy values of 100%, 95.65%, and 93.62% for reactions containing 10,000, 1000, and 100 copies of SARS-CoV-2 genetic material, respectively.

We then evaluated the feasibility of performing LAMP in saliva samples spiked with synthetic SARS-CoV-2 genetic material.

### 3.3. Assessment of Sensitivity and Specificity Using Saliva Samples

Nasopharyngeal sampling is an effective but intrusive and unpleasant test procedure for the person being tested [28] and needs to be performed by trained personnel. Therefore, the use of saliva samples rather than nasopharyngeal samples greatly adds to the concept of a testing strategy amenable to widespread implementation [11,12]. Moreover, several recent contributions have reported similar or higher SARS-CoV-2 loads in saliva than in nasopharyngeal samples [17,18,29].

Human saliva is a complex fluid [30] and, therefore, poses challenges for direct molecular diagnostics, mainly due to its high viscosity and broad chemical composition. These challenges can be addressed by the use of virus transport media [31]. Diluting the saliva in transport solutions reduces the effective viscosity of the sample, while the transport media themselves typically contain nuclease deactivators and RNA stabilizers that enable the long-distance transportation of samples to centralized labs. However, this inactivation step reduces the probability of successful diagnosis because it dilutes the viral load of the sample.

To overcome this issue, we introduced a simple saliva concentration step based on the use of a laminated PES membrane device (Figure 2 and Figure 3). PES membranes have been used before in applications related to the amplification of genetic material, and PES is known not to interfere with or inhibit amplification [32,33,34,35]. In the present application, the use of laminated PES membranes enables an extraction-free diagnosis while eliminating the need to dilute the saliva sample with virus transport media.

Concentration using laminated PES membranes serves a second important purpose in this portable SARS-CoV-2 diagnostic method, related to the analysis of acidic saliva samples. In the case of colorimetric amplification schemes, such as the LAMP method that we use here, acidic saliva severely interferes with the discrimination ability of the test. In colorimetric LAMP, discrimination between positive and negative samples is based on the change in pH induced in a weakly buffered reaction mix by the release of H+ ions during amplification and the consequent change in color across the threshold of phenol red (pH ~6.8). Initially, acidic saliva samples may induce a change in color in negative samples and provoke false positives [36]. In addition, large volumes of neutral or slightly alkaline saliva samples are not compatible with colorimetric LAMP. In our experiments, we observed that saliva samples spiked with synthetic SARS-CoV-2 nucleic acids compromised the amplification if more than 2 µL of saliva were added to 20 µL of the LAMP reaction mix.

The use of laminated PES membrane devices serves the purpose of removing a significant fraction of the liquid volume, thereby attenuating both the effect of the saliva sample pH on the original pH and the diluting effect of large volumes of saliva.

Based on geometric arguments, the volume of a drop of saliva, as dispensed with a disposable 100 µL pipette, ranges between 10 and 25 µL, as calculated from experiments conducted with different saliva samples (*n* = 20) (Figure 3A,B). The volume of liquid absorbed in the micro-well of the filter paper device is 2.4 ± 0.2 µL (Figure 3C). Then, by the absorption of the excess liquid in the PES membrane, this technique reduces the working volume by approximately one order of magnitude. Remarkably, the genetic material (and proteins) present in the saliva sample are retained mostly in the micro-well section, thereby providing a straightforward alternative to concentration. Our experiments suggest a retention of 80% ± 5% of the nucleic acids contained in the original drop of saliva in the micro-well area.

We spiked human saliva with synthetic SARS-CoV-2 genetic material and a fluorescent nucleic acid intercalant (Figure 3D) and then placed drops of this saliva onto filter paper strips. Figure 3E visually shows that the fluorescently labeled nucleic acid remains mainly confined in the micro-well region in which the saliva drop was originally placed.

The concentration of nucleic acids in the micro-well zone was an unexpected result. We anticipated that the laminated paper strips would have acted like paper chromatography devices [37] and that a defined band of nucleic acids would be evident at a zone somewhere between the deposition in the micro-well and the distal extreme of the strip.

However, our results demonstrate that nucleic acids are effectively concentrated within the micro-well circumference. We hypothesize that the electrostatic/repulsive forces induced in the laminated plastic during lamination overcome capillarity and define a circular barrier for the migration of large and charged molecules. To test this hypothesis, we simulated the electrostatic force field acting on negatively charged genetic material within the laminated device. For this, we assumed a certain distribution of charges in the laminated device as described in the Materials and Methods (Figure 3F). The simulation results suggest that a strong accumulation of charges in the perimeter of the circular section of the exposed PES (red rim in Figure 3F) may be responsible for the retention of nucleic acids in the micro-well. We repeated this experiment using saliva samples spiked with RNA extracts from volunteers diagnosed as COVID-19 (+) by RT-qPCR. Similarly, the genetic material mainly remained confined within the micro-well of the laminated filter paper device.

We cut 5 cm long filter paper strips into six different sections and analyzed the nucleic acid content by spectrophotometry using a Nanodrop instrument. On average, 82% of the genetic material remained in the micro-well section of the device (Figure 3F). In addition, we ran extraction-free and final-point PCR experiments to amplify SARS-CoV-2 genetic material from seven different sections of the filter paper strips (Figure 3G). The expected amplification products, as revealed by gel electrophoresis, were only observed in the lanes corresponding to amplification products eluted from sections A and B of the laminated filter paper devices. These sections correspond to the micro-well location and its neighboring section.

These results demonstrated that nucleic acid concentration is in fact feasible using simple laminated filter paper strips and that this genetic material is amenable to further amplification. In turn, this suggests that the solids concentrated from saliva do not inhibit nucleic acid amplification.

### 3.4. Limits of Detection in Saliva Samples Spiked with SARS-CoV-2 RNA

We conducted a series of LAMP reactions to detect and amplify SARS-CoV-2 from sections of the PES membrane containing concentrated nucleic acids from saliva samples spiked with RNA extracts from positive patients. After concentration of the inactivated saliva in laminated paper strips, the micro-well section was cut and added to the colorimetric LAMP reaction mix. In these experiments, we used the colorimetric LAMP reaction mix and incubated at 62 °C for 35 min. A selectivity of 100% was observed among 20 saliva samples spiked with 300 ng of RNA extracts.

**Figure 4:**
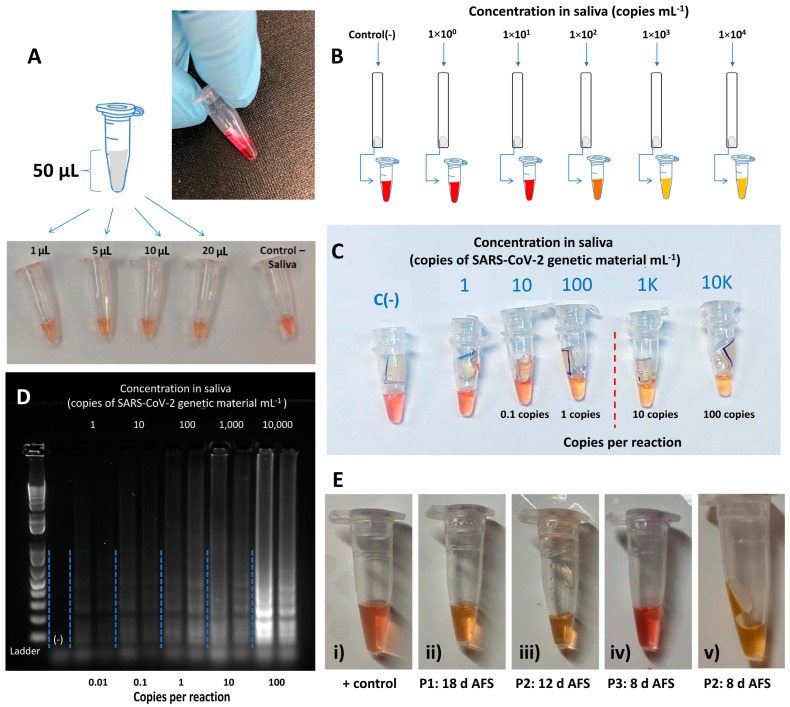
Concentration and amplification of SARS-CoV-2 RNA from patient samples using PES membranes. (**A**) Saliva samples were spiked with SARS-CoV-2 RNA extracts from COVID-19 positive patients, and different volumes of the eluted material (1, 2, 5, 10, and 20 µL) were added to Eppendorf tubes containing reaction mix and primers. In all cases, the LAMP proceeded, as indicated by the red-to-yellow change in color. (**B**) Schematic representation of an experimental design to calculate the limit of detection of LAMP following concentration of saliva samples in laminated PES membranes. Saliva samples were spiked with different concentrations of synthetic SARS-CoV-2 DNA and added to laminated PES membranes to concentrate the genetic material. (**C**) LAMP of the genetic material retained in the circular region of the PES strips. The results suggest that samples initially containing 1 × 10^3^ copies of SARS-CoV-2 genetic material per mL of saliva (equivalent to 10 copies of SARS-CoV-2 genetic material per reaction) can be clearly discriminated from negative samples. (**D**) Gel electrophoresis of the product of amplification of LAMP reactions containing different copy numbers of genetic material of SARS-CoV-2 as concentrated using PES strips. (**E**) Images of saliva samples after LAMP: color of the LAMP reaction mix after incubation of samples containing 2 µL of saliva from (**i**) a COVID-19 (−patient (negative control); (**ii**–**iv**) three different COVID-19(+) patients (referred to as P1, P2, and P3) within the same household, and (**v**) color of the LAMP reaction mix after incubation of a sample of saliva from patient P3. The sample was first concentrated in a PES device. Labels indicate the time of performance of the assay after first symptom (AFS).

Figure 4A shows the results from an experiment in which 300 ng of RNA extract was diluted in 2 µL of saliva and added to 48 µL of phosphate-buffered saline (PBS).

This mix was concentrated using PES membrane devices, the micro-well section was cut out, and its contents were eluted into 50 µL of PBS. Different volumes of the eluted material (i.e., 1, 2, 5, 10, and 20 µL) were added to Eppendorf tubes containing the reaction mix and primers. In all cases, LAMP proceeded, as indicated by the red-to-yellow change in color (Figure 4A).

To establish the limit of detection of these LAMP variants, we conducted an additional series of experiments, spiking saliva with synthetic SARS-CoV-2 genetic material at different concentrations. To perform this, we prepared dilutions containing 1, 10, 100, 1000, and 10,000 synthetic copies per mL of saliva.

These saliva samples were inactivated (95 °C for 5 min) and concentrated in laminated paper strips. Figure 4B shows images from a typical experiment. Consistently, the tubes spiked with 10,000 and 1000 synthetic copies of SARS-CoV-2 per mL of saliva changed from red to yellow in less than 30 min. Consistently, as the concentration of target nucleic acid copies decreased, the probability of successful amplification also decreased. Tubes that contained samples spiked with 100 copies of SARS-CoV-2 mL^−1^ of saliva changed from red to yellow 70% of the time (*n* = 10), and tubes spiked with 10 copies of SARS-CoV-2 mL^−1^ of saliva changed from red to yellow 50% of the time (*n* = 10). This set of results suggests that a limit of detection of 1000 viral copies mL^−1^ of saliva can be reliably established (Figure 4C). This limit of detection is equivalent to 10 copies of SARS-CoV-2 genetic material per reaction. To calculate the number of copies per reaction, we considered that a 10 µL saliva sample can be absorbed by the PES strips (as estimated experimentally) (Figure 3B), and that nucleic acids remain confined in the first portion of the PES strips (as shown in Figure 3G).

We corroborated the amplification by visualizing LAMP products using gel electrophoresis for the different viral loads we tested (Figure 4D). We were able to generate a visible array of bands of amplification products, a typical signature of LAMP, across the range of synthetic viral loads from 1 to 10,000 copies mL^−1^.

### 3.5. Testing at Home: Results from a Pilot Study

From July 2020 to February 2021, reagents and kits were deployed to 10 different locations (mainly private homes) in the metropolitan area of the City of Monterrey in México to enable the presumptive diagnostics of 28 samples from 23 different volunteers. Most volunteers (15/23) exhibited COVID-19-related symptoms or had been in contact with diagnosed COVID-19 patients (2/23) (Table 2). In addition, we diagnosed samples from 11 asymptomatic volunteers who had tested negative for COVID-19 in RT-qPCR assays.

From this reduced set of home-made diagnostic assays, a high sensitivity (i.e., 100%) and specificity (i.e., 87.50%) may be inferred. We presumably identified 12 positive samples from 9 volunteers who later tested positive by standard RT-qPCR (i.e., sensitivity of 100%). Three COVID-19(+) volunteers provided two different samples taken three days apart. We presumably identified 16 samples as negative by RT-PCR. Two of these samples tested positive by LAMP. That is, we observed disagreement in only two samples diagnosed as positive using our kit, and which were seen as negative by RT-qPCR.

In addition, our experimental results, based on a limited number of samples from COVID-19 patients, suggest that the process of concentration using PES membranes before LAMP enables a clearer discrimination between positive and negative results. Figure 4E compares the final color of the reaction mix containing saliva samples from three patients diagnosed as COVID-19(+) by RT-PCR and living in the same household. These samples were assayed both with and without the PES membrane concentration step. All three samples changed color after 30 min of incubation when the PES membrane was used to concentrate the saliva samples. By contrast, only two of the three samples changed color from red to yellow when 2 µL of inactivated saliva was directly added to the reaction mix and incubated for 30 min (Figure 4E).

These sets of results suggest that concentrating using PES membrane devices could enhance the sensitivity of the assay. Of note, our results also suggest that this self-diagnosis method may be used for effective identification of the presence of SARS-CoV-2 genetic material in saliva from real patients during the first 2–14 days of infection (from the onset of symptoms).

Overall, we observed a sensitivity of 100% and a specificity of 87% based on the limited number of actual saliva samples that we used (n_COVID-19(+)_ = 12; n_COVID-19(_−_)_ = 15). The overall accuracy of the test is estimated at 92.86% (*n* = 28) with patients themselves, instead of expert qualified personnel, running the experiments.

## 4. Discussion

Intensifying and democratizing the testing effort has been clearly recognized as a key strategy for controlling the COVID-19 pandemic [38,39,40]. Nevertheless, the limitations of the diagnostic strategies available for conducting widespread diagnostics became clear during the COVID-19 pandemic. In this context, the development of simple, portable, and cost-effective diagnostic systems for COVID-19 is an urgent need.

Recently, several groups have shown that accurate discrimination between COVID-19(+) and COVID-19(−) samples is possible using extraction-free RT-qPCR (i.e., average sensitivity and selectivity above 91% and 99%, respectively) [27,41]. High sensitivity and selectivity values have also been reported in extraction-free colorimetric LAMP implementations conducted using saliva samples (i.e., average sensitivity and selectivity values higher than 85% and 95%, respectively) [14,20,42]. In these protocols, inactivation and extraction are frequently mediated by heat treatment. Smyrlaki et al. extensively investigated the performance of an extraction-free RT-qPCR protocol in which saliva or nasopharyngeal samples were heated at 95 °C for 5 min for virus inactivation and then directly amplified [27]. Lalli et al. showed successful amplification using a similar colorimetric LAMP protocol following heating of saliva samples at 64 °C for 30 min and then at 95 °C for 5 min, and optionally adding protease K [14]. Recently, Hemati et al. found that heating at 60 °C for 30 min improved the RNA quantity and quality for RT-qPCR amplification experiments [43]. To preserve the simplicity of the method, we decided to inactivate exclusively by a heat treatment at 95 °C for 5 min [27,41] or at 60 °C for 30 min [41] in the experiments reported here. Both of these inactivation methods yielded similar results in our limited experimental pool. All samples diagnosed as SARS-CoV-2(+) by PCR were diagnosed as positive by our LAMP-based method, regardless of the inactivation method used. We observed two false positives: one associated with the high temperature and short time inactivation protocol. Indeed, general evidence indicates that the inclusion of protease K in extraction-free protocols improves performance, which is generally measured as a decrease in the cycle threshold (CT) in RT-PCR methods or as an improvement in the limits of detection in LAMP-based studies [14,44]. The addition of protease K or trypsin [45] to optimize extraction should be explored in future studies.

Taken together, these results suggest that several extraction-free implementations of amplification methods, including colorimetric LAMP, can successfully identify COVID-19 positive patients from their nasopharyngeal and saliva samples. However, the demonstration of deployable, extraction-free, and high accuracy SARS-CoV-2 testing from saliva remains elusive, and only a few reports are available on this topic [19,43,46,47,48]. Here, we investigated the performance of an extraction-free colorimetric LAMP method assisted by a stage of concentration of nucleic acids in laminated PES membrane devices from saliva samples. We found that the concentration stage in the laminated paper lowers the limit of detection of SARS-CoV-2 to 1000 copies mL^−1^ of saliva, making it competitive with (or maybe improved over) extraction-free RT-qPCR by at least one order of magnitude [49]. This LOD falls within the range reported previously for extraction-free LAMP-based methods. For example, Lali et al. [14] and Rivas-Macho et al. [42] reported LOF values of approximately 60 viral copies per reaction for an extraction-free colorimetric LAMP-based method conducted using simulated saliva samples. We previously reported a limit of detection of 625 copies per reaction for a colorimetric LAMP-based method using the same primer set used in the present paper, but not assisted by PES strips. The RT-PCR protocols conducted using nasopharyngeal samples and assisted by nucleic acid extraction still exhibit better LODs than extraction-free LAMP-based methods (i.e., between 10 to 500 copies per mL of sample [50] versus 100 to 1000 copies per mL of saliva).

Other strategies have been reported for the simplification of the separation/concentration of SARS-CoV-2 nucleic acids from saliva while avoiding convectional lab extraction protocols [51,52]. These strategies, often based on the use of silica particles or carbon nanotubes, achieve limits of detection of approximately 5 × 10^3^ [52] to 10 × 10^3^ [51] copies mL^−1^. The limit of detection of our method falls within this range (i.e., approximately 1 × 10^3^ copies mL^−1^) and arguably uses a simpler methodology (i.e., without a centrifugation step).

In the context of the COVID-19 pandemic, the method described here competes with other molecular methods in terms of accuracy while surpassing RT-qPCR in cost effectiveness. While the market value of a traditional RT-qPCR apparatus (the current gold standard for COVID-19 diagnostics) is in the range of 10,000 to USD 40,000, the cost of the DIY incubator presented here is about USD 20. These differences in capital investment between conventional RT-qPCR and colorimetric LAMP are significant, especially during an epidemic or pandemic crisis when a rational investment of resources is critical. While the quantitative capabilities of testing using an RT-qPCR platform are undisputable, the capacity of many countries to rapidly, effectively, and massively establish diagnostic centers based on RT-qPCR has proven questionable. The pandemic scenarios experienced in India, México, and Malaysia, among many others, have crudely demonstrated that centralized labs are not an ideal solution during emergencies. Portable diagnostic systems may provide vital flexibility and a speed of response that RT-qPCR platforms cannot deliver.

In this work, we took a step further and experimentally explored the feasibility of deploying COVID-19 kits for self-diagnostics (Figure 1B). This will further decrease the cost of operation in comparison to approaches of using RT-qPCR apparatus, which must be operated by highly trained personnel. To our knowledge, this is the first experimental pilot study that explores this option as a radically different alternative to the typical centralized system of molecular diagnostics prevailing during the COVID-19 pandemic. The use of molecular self-diagnostics [53] was recently suggested (but not experimentally explored) in the context of antigen-based testing in Nigeria. The authors conducted a survey among nearly 60 volunteers to identify the element of perception towards the implementation of self-testing for the detection of SARS-CoV-2 infections among the Nigerian population. The survey presented self-testing as a feasible alternative to increase, in a cost-effective fashion, the low rate of COVID-19 testing in Nigeria. The results of this pilot study suggest that distributing personal diagnostic kits for home-made self-diagnostics is feasible. This is an entirely different scheme of testing based on decentralization and parallelization. However, this study was performed with a limited number of volunteers (*n* = 23). A larger study must be conducted to demonstrate the feasibility of the widespread deployment of this distributed testing effort and to fully assess its benefits.

## 5. Conclusions

The limitations of the available diagnostic strategies to conduct widespread diagnostics have become clear during the COVID-19 pandemic. Ideally, diagnostics should be cost-effective, feasible, and reliable at home, thereby empowering every citizen with the ability to continuously verify their own health.

Here, we describe the fabrication of a portable DIY incubator that enables the thermal inactivation of saliva samples and LAMP-based diagnostics of COVID-19 at home. In addition, we introduced the use of a simple laminated PES membrane device for the concentration of nucleic acids from saliva samples for conducting LAMP at home.

Using the methods described here, we were able to consistently detect the presence of SARS-CoV-2 synthetic nucleic acids in saliva samples. We show that, after only 30 min of incubation in a simple DIY Eppendorf tube incubator, samples containing a viral load in the range of 10^4^ to 10^2^ copies could be clearly discriminated from negative samples by visual inspection with the naked eye at home.

LAMP-based methods, such as the one introduced here, may have an intermediate cost effectiveness—somewhere between RT-qPCR methods, which are more accurate but less user-friendly and suitable to decentralization, and LFIA-based methods, which also enable decentralized testing at lower costs than LAMP-based methods but still with higher limits of detection (in general, at least one order of magnitude higher) [5,6]. The LAMP-based methods may also offer greater reliability than molecular-based assays [5,6].

Taken together, this evidence suggests that extraction-free colorimetric LAMP provides a means of cost-effective and massive diagnostics of SARS-CoV-2 and represents a promising tool for pandemic containment that deserves further exploration.

## Figures and Tables

**Table 1:** Primer sequences used in LAMP experiments. Two different sets of primers were used, each directed at the RNA sequence encoding the N sequence of SARS-CoV-2.

Set	Description	Primers Sequence (5′ > 3′)
Primer set β	2019-nCoV 2-F3	CCAGAATGGAGAACGCAGTG
	2019-nCoV 2-B3	CCGTCACCACCACGAATT
	2019-nCoV 2-FIP	AGCGGTGAACCAAGACGCAGGGCGCGATCAAAACAACG
	2019-nCoV 2-BIP	AATTCCCTCGAGGACAAGGCGAGCTCTTCGGTAGTAGCCAA
	2019-nCoV 2-LF	TTATTGGGTAAACCTTGGGGC
	2019-nCoV 2-LB	TAACACCAATAGCAGTCCAGATGA

**Table 2:** Summary of LAMP and RT-qPCR diagnostic results for saliva samples from COVID-19 positive and negative volunteers, as diagnosed by RT-qPCR.

Sample Code	LAMP	PCR	Days from Onset of Symptoms	Agreement (+)	Agreement (−)	Overall Agreement
S01	1	1	2	1	0	1
S02-1	1	1	2	1	0	1
S03	1	1	2	1	0	1
S04-1	1	1	12	1	0	1
S05	1	1	18	1	0	1
S06	1	1	8	1	0	1
S07-1	1	1	5	1	0	1
S08-1	1	1	4	1	0	1
S09-1	1	1	3	1	0	1
S07-2	1	1	11	1	0	1
S08-2	1	1	10	1	0	1
S09-2	1	1	3	1	0	1
S10	0	0	4	0	1	1
S11	0	0	2	0	1	1
S12	1	0	c	0	0	0
S13	0	0	c	0	1	1
S04-0	0	0	-	0	1	1
S14	0	0	-	0	1	1
S02-0	0	0	-	0	1	1
S15	0	0	-	0	1	1
S16	0	0	-	0	1	1
S17	0	0	-	0	1	1
S18	0	0	-	0	1	1
S19	0	0	-	0	1	1
S20	0	0	-	0	1	1
S21	0	0	-	0	1	1
S22	0	0	-	0	1	1
S32	1	0	5	0	0	0
	14	12		12	14	26

## Data Availability

The data presented in this study are openly available upon request.

## References

[1] Sharfstein J.M., Becker S.J., Mello M.M. (2020). Diagnostic Testing for the Novel Coronavirus. JAMA J. Am. Med. Assoc..

[2] Younes N., Al-Sadeq D.W., AL-Jighefee H., Younes S., Al-Jamal O., Daas H.I., Yassine H.M., Nasrallah G.K. (2020). Challenges in Laboratory Diagnosis of the Novel Coronavirus SARS-CoV-2. Viruses.

[3] Esbin M.N., Whitney O.N., Chong S., Maurer A., Darzacq X., Tjian R. (2020). Overcoming the Bottleneck to Widespread Testing: A Rapid Review of Nucleic Acid Testing Approaches for COVID-19 Detection. RNA.

[4] Hsieh W.Y., Lin C.H., Lin T.C., Lin C.H., Chang H.F., Tsai C.H., Wu H.T., Lin C.S. (2021). Development and Efficacy of Lateral Flow Point-of-Care Testing Devices for Rapid and Mass COVID-19 Diagnosis by the Detections of SARS-CoV-2 Antigen and Anti-SARS-CoV-2 Antibodies. Diagnostics.

[5] Stanley S., Hamel D.J., Wolf I.D., Riedel S., Dutta S., Contreras E., Callahan C.J., Cheng A., Arnaout R., Kirby J.E. (2022). Limit of Detection for Rapid Antigen Testing of the SARS-CoV-2 Omicron and Delta Variants of Concern Using Live-Virus Culture. J. Clin. Microbiol..

[6] Cubas-Atienzar A.I., Kontogianni K., Edwards T., Wooding D., Buist K., Thompson C.R., Williams C.T., Patterson E.I., Hughes G.L., Baldwin L. (2021). Limit of Detection in Different Matrices of 19 Commercially Available Rapid Antigen Tests for the Detection of SARS-CoV-2. Sci. Rep..

[7] Cheng M.P., Papenburg J., Desjardins M., Kanjilal S., Quach C., Libman M., Dittrich S., Yansouni C.P. (2020). Diagnostic Testing for Severe Acute Respiratory Syndrome-Related Coronavirus 2: A Narrative Review. Ann. Intern. Med..

[8] Vogels C.B.F., Brito A.F., Wyllie A.L., Fauver J.R., Ott I.M., Kalinich C.C., Petrone M.E., Casanovas-Massana A., Catherine Muenker M., Moore A.J. (2020). Analytical Sensitivity and Efficiency Comparisons of SARS-CoV-2 RT–QPCR Primer–Probe Sets. Nat. Microbiol..

[9] Giri A.K., Rana D.R. (2020). Charting the Challenges behind the Testing of COVID-19 in Developing Countries: Nepal as a Case Study. Biosaf. Health.

[10] Tran N.K., Albahra S., Rashidi H., May L. (2023). Innovations in Infectious Disease Testing: Leveraging COVID-19 Pandemic Technologies for the Future. Clin. Biochem..

[11] Hamid H., Khurshid Z., Adanir N., Zafar M.S., Zohaib S. (2020). COVID-19 Pandemic and Role of Human Saliva as a Testing Biofluid in Point-of-Care Technology. Eur. J. Dent..

[12] Yee R., Truong T.T., Pannaraj P.S., Eubanks N., Gai E., Jumarang J., Turner L., Peralta A., Lee Y., Dien Bard J. (2021). Saliva Is a Promising Alternative Specimen for the Detection of SARS-CoV-2 in Children and Adults. Am. Soc. Microbiol..

[13] Rose D., Ranoa E., Holland R.L., Alnaji F.G., Green K.J., Wang L., Brooke C.B., Burke M.D., Fan T.M., Hergenrother P.J. (2020). Saliva-Based Molecular Testing for SARS-CoV-2 That Bypasses RNA Extraction. biorxiv.

[14] Lalli M.A., Langmade J.S., Chen X., Fronick C.C., Sawyer C.S., Burcea L.C., Wilkinson M.N., Fulton R.S., Heinz M., Buchser W.J. (2021). Rapid and Extraction-Free Detection of SARS-CoV-2 from Saliva by Colorimetric Reverse-Transcription Loop-Mediated Isothermal Amplification. Clin. Chem..

[15] Azzi L., Maurino V., Baj A., Dani M., d’Aiuto A., Fasano M., Lualdi M., Sessa F., Alberio T. (2021). Diagnostic Salivary Tests for SARS-CoV-2. J. Dent. Res..

[16] Tahir B., Weldegebreal F., Ayele F., Ayana D.A. (2023). Comparative Evaluation of Saliva and Nasopharyngeal Swab for SARS-CoV-2 Detection Using RT-QPCR among COVID-19 Suspected Patients at Jigjiga, Eastern Ethiopia. PLoS ONE.

[17] Wyllie A.L., Fournier J., Casanovas-Massana A., Campbell M., Tokuyama M., Vijayakumar P., Warren J.L., Geng B., Muenker M.C., Moore A.J. (2020). Saliva or Nasopharyngeal Swab Specimens for Detection of SARS-CoV-2. N. Engl. J. Med..

[18] Teo A.K.J., Choudhury Y., Tan I.B., Cher C.Y., Chew S.H., Wan Z.Y., Cheng L.T.E., Oon L.L.E., Tan M.H., Chan K.S. (2021). Saliva Is More Sensitive than Nasopharyngeal or Nasal Swabs for Diagnosis of Asymptomatic and Mild COVID-19 Infection. Sci. Rep..

[19] Alvarez M.M., Bravo-González S., González-González E., Santiago G.T. (2021). De Portable and Label-Free Quantitative Loop-Mediated Isothermal Amplification (Lf-Qlamp) for Reliable COVID-19 Diagnostics in Three Minutes of Reaction Time: Arduino-Based Detection System Assisted by a Ph Microelectrode. Biosensors.

[20] González-González E., Lara-Mayorga I.M., Rodríguez-Sánchez I.P., Zhang Y.S., Martínez-Chapa S.O., Santiago G.T., Alvarez M.M. (2021). Colorimetric Loop-Mediated Isothermal Amplification (LAMP) for Cost-Effective and Quantitative Detection of SARS-CoV-2: The Change in Color in LAMP-Based Assays Quantitatively Correlates with Viral Copy Number. Anal. Methods.

[21] Kitajima H., Tamura Y., Yoshida H., Kinoshita H., Katsuta H., Matsui C., Matsushita A., Arai T., Hashimoto S., Iuchi A. (2021). Clinical COVID-19 Diagnostic Methods: Comparison of Reverse Transcription Loop-Mediated Isothermal Amplification (RT-LAMP) and Quantitative RT-PCR (QRT-PCR). J. Clin. Virol..

[22] Zou H., Zhang Y., Guo L., Wang P., He X., Dai G., Zheng H., Chen C., Wang A.C., Xu C. (2019). Quantifying the Triboelectric Series. Nat. Commun..

[23] Breite D., Went M., Thomas I., Prager A., Schulze A. (2016). Particle Adsorption on a Polyether Sulfone Membrane: How Electrostatic Interactions Dominate Membrane Fouling. RSC Adv..

[24] Kaarj K., Akarapipad P., Yoon J.Y. (2018). Simpler, Faster, and Sensitive Zika Virus Assay Using Smartphone Detection of Loop-Mediated Isothermal Amplification on Paper Microfluidic Chips. Sci. Rep..

[25] Tanner N.A., Zhang Y., Evans T.C. (2015). Visual Detection of Isothermal Nucleic Acid Amplification Using PH-Sensitive Dyes. Biotechniques.

[26] Rusk N. (2011). Torrents of Sequence. Nat. Methods.

[27] Smyrlaki I., Ekman M., Lentini A., Rufino de Sousa N., Papanicolaou N., Vondracek M., Aarum J., Safari H., Muradrasoli S., Rothfuchs A.G. (2020). Massive and Rapid COVID-19 Testing Is Feasible by Extraction-Free SARS-CoV-2 RT-PCR. Nat. Commun..

[28] Ali F., Sweeney D.A. (2020). No One Likes a Stick up Their Nose: Making the Case for Saliva-Based Testing for Coronavirus Disease 2019 (COVID-19). Clin. Infect. Dis..

[29] Babady N.E., McMillen T., Jani K., Viale A., Robilotti E.V., Aslam A., Diver M., Sokoli D., Mason G., Shah M.K. (2021). Performance of Severe Acute Respiratory Syndrome Coronavirus 2 Real-Time RT-PCR Tests on Oral Rinses and Saliva Samples. J. Mol. Diagn..

[30] Sahajpal N.S., Mondal A.K., Njau A., Ananth S., Ghamande S., Hegde M., Chaubey A., Rojiani A.M., Kolhe R. (2021). COVID-19 Screening in a Healthcare or Community Setting: Complexity of Saliva as a Specimen for PCR-Based Testing. Future Med. Chem..

[31] Toppings N., Mohon A., Lee Y., Kumar H., Lee D., Kapoor R., Singh G., Oberding L., Abdullah O., Kim K. (2021). Saliva-Dry LAMP: A Rapid Near-Patient Detection System for SARS-CoV-2. Sci. Rep..

[32] Rodriguez N.M., Wong W.S., Liu L., Dewar R., Klapperich C.M. (2016). A Fully Integrated Paperfluidic Molecular Diagnostic Chip for the Extraction, Amplification, and Detection of Nucleic Acids from Clinical Samples. Lab Chip.

[33] Batule B.S., Seok Y., Kim M.G. (2020). Paper-Based Nucleic Acid Testing System for Simple and Early Diagnosis of Mosquito-Borne RNA Viruses from Human Serum. Biosens. Bioelectron..

[34] Linnes J.C., Rodriguez N.M., Liu L., Klapperich C.M. (2016). Polyethersulfone Improves Isothermal Nucleic Acid Amplification Compared to Current Paper-Based Diagnostics. Biomed. Microdevices.

[35] Seok Y., Joung H.A., Byun J.Y., Jeon H.S., Shin S.J., Kim S., Shin Y.B., Han H.S., Kim M.G. (2017). A Paper-Based Device for Performing Loop-Mediated Isothermal Amplification with Real-Time Simultaneous Detection of Multiple DNA Targets. Theranostics.

[36] Uribe-Alvarez C., Lam Q., Baldwin D.A., Chernoff J. (2021). Low Saliva PH Can Yield False Positives Results in Simple RT-LAMP-Based SARS-CoV-2 Diagnostic Tests. PLoS ONE.

[37] Sullivan B.P., Bender A.T., Ngyuen D.N., Zhang J.Y., Posner J.D. (2021). Nucleic Acid Sample Preparation from Whole Blood in a Paper Microfluidic Device Using Isotachophoresis. J. Chromatogr. B.

[38] Alvarez M.M., González-González E., Trujillo-de Santiago G. (2021). Modeling COVID-19 Epidemics in an Excel Spreadsheet to Enable First-Hand Accurate Predictions of the Pandemic Evolution in Urban Areas. Sci. Rep..

[39] Choi W., Shim E. (2021). Optimal Strategies for Social Distancing and Testing to Control COVID-19. J. Theor. Biol..

[40] Alvarez M.M., Bravo-González S., Trujillo-De Santiago G. (2021). Modeling Vaccination Strategies in an Excel Spreadsheet: Increasing the Rate of Vaccination Is More Effective than Increasing the Vaccination Coverage for Containing COVID-19. PLoS ONE.

[41] Allicock O.M., Yolda-Carr D., Earnest R., Breban M.I., Vega N., Ott I.M., Kalinich C., Alpert T., Petrone M.E., Wyllie A.L. (2023). Method Versatility in RNA Extraction-Free PCR Detection of SARS-CoV-2 in Saliva Samples. Prog. Biophys. Mol. Biol..

[42] Rivas-Macho A., Sorarrain A., Marimón J.M., Goñi-de-Cerio F., Olabarria G. (2023). Extraction-Free Colorimetric RT-LAMP Detection of SARS-CoV-2 in Saliva. Diagnostics.

[43] Hemati M., Soosanabadi M., Ghorashi T., Ghaffari H., Vahedi A., Sabbaghian E., Nejad Z.R., Salati A., Danaei N., Kokhaei P. (2021). Thermal Inactivation of COVID-19 Specimens Improves RNA Quality and Quantity. J. Cell. Physiol..

[44] Genoud V., Stortz M., Waisman A., Berardino B.G., Verneri P., Dansey V., Salvatori M., Lenicov F.R., Levi V. (2021). Extraction-Free Protocol Combining Proteinase K and Heat Inactivation for Detection of SARS-CoV-2 by RT-QPCR. PLoS ONE.

[45] García-Sorribes S., Lara-Hernández F., Manzano-Blasco I., Abadía-Otero J., Albert E., Mulet A., Briongos-Figuero L.S., Gabella-Martín M., Torres I., Signes-Costa J. (2023). Sample Treatment with Trypsin for RT-LAMP COVID-19 Diagnosis. Biology.

[46] Ning B., Yu T., Zhang S., Huang Z., Tian D., Lin Z., Niu A., Golden N., Hensley K., Threeton B. (2021). A Smartphone-Read Ultrasensitive and Quantitative Saliva Test for COVID-19. Sci. Adv..

[47] Kobayashi G.S., Brito L.A., Moreira D.d.P., Suzuki A.M., Hsia G.S.P., Pimentel L.F., de Paiva A.P.B., Dias C.R., Lourenço N.C.V., Oliveira B.A. (2021). A Novel Saliva RT-LAMP Workflow for Rapid Identification of COVID-19 Cases and Restraining Viral Spread. Diagnostics.

[48] Howson E.L.A., Kidd S.P., Armson B., Goring A., Sawyer J., Cassar C., Cross D., Lewis T., Hockey J., Rivers S. (2021). Preliminary Optimisation of a Simplified Sample Preparation Method to Permit Direct Detection of SARS-CoV-2 within Saliva Samples Using Reverse-Transcription Loop-Mediated Isothermal Amplification (RT-LAMP). J. Virol. Methods.

[49] Byrnes S.A., Gallagher R., Steadman A., Bennett C., Rivera R., Ortega C., Motley S.T., Jain P., Weigl B.H., Connelly J.T. (2021). Multiplexed and Extraction-Free Amplification for Simplified SARS-CoV-2 RT-PCR Tests. Anal. Chem..

[50] Fung B., Gopez A., Servellita V., Arevalo S., Ho C., Deucher A., Thornborrow E., Chiu C., Miller S. (2020). Direct Comparison of SARS-CoV-2 Analytical Limits of Detection across Seven Molecular Assays. J. Clin. Microbiol..

[51] Jeong S., González-Grandío E., Navarro N., Pinals R.L., Ledesma F., Yang D., Landry M.P. (2021). Extraction of Viral Nucleic Acids with Carbon Nanotubes Increases SARS-CoV-2 Quantitative Reverse Transcription Polymerase Chain Reaction Detection Sensitivity. ACS Nano.

[52] Rabe B.A., Cepko C. (2020). SARS-CoV-2 Detection Using Isothermal Amplification and a Rapid, Inexpensive Protocol for Sample Inactivation and Purification. Proc. Natl. Acad. Sci. USA.

[53] Undelikwo V.A., Shilton S., Folayan M.O., Alaba O., Reipold E.I., Martínez-Pérez G.Z. (2023). COVID-19 Self-Testing in Nigeria: Stakeholders’ Opinions and Perspectives on Its Value for Case Detection. PLoS ONE.

